# Affinity Purification of Human Factor H on Polypeptides Derived from Streptococcal M Protein: Enrichment of the Y402 Variant

**DOI:** 10.1371/journal.pone.0081303

**Published:** 2013-11-21

**Authors:** O. Rickard Nilsson, Jonas Lannergård, B. Paul Morgan, Gunnar Lindahl, Mattias C. U. Gustafsson

**Affiliations:** 1 Department of Laboratory Medicine, Lund University, Lund, Sweden; 2 Department of Veterinary Disease Biology, University of Copenhagen, Copenhagen, Denmark; 3 Institute of Infection & Immunity, Cardiff University, Cardiff, United Kingdom; Montana State University, United States of America

## Abstract

Recent studies indicate that defective activity of complement factor H (FH) is associated with several human diseases, suggesting that pure FH may be used for therapy. Here, we describe a simple method to isolate human FH, based on the specific interaction between FH and the hypervariable region (HVR) of certain *Streptococcus pyogenes* M proteins. Special interest was focused on the FH polymorphism Y402H, which is associated with the common eye disease age-related macular degeneration (AMD) and has also been implicated in the binding to M protein. Using a fusion protein containing two copies of the M5-HVR, we found that the Y402 and H402 variants of FH could be efficiently purified by single-step affinity chromatography from human serum containing the corresponding protein. Different M proteins vary in their binding properties, and the M6 and M5 proteins, but not the M18 protein, showed selective binding of the FH Y402 variant. Accordingly, chromatography on a fusion protein derived from the M6-HVR allowed enrichment of the Y402 protein from serum containing both variants. Thus, the exquisite binding specificity of a bacterial protein can be exploited to develop a simple and robust procedure to purify FH and to enrich for the FH variant that protects against AMD.

## Introduction

Factor H (FH) plays a key role in the regulation of complement activation, both in the fluid phase and on cell surfaces [1-3]. This ~150 kDa glycoprotein, which is composed of 20 short consensus repeats (SCRs) and occurs at a serum concentration of ~250 µg/ml [4], exhibits polymorphisms and mutations that are associated with human diseases, including the common eye disease age-related macular degeneration (AMD) and the rare kidney disease atypical hemolytic uremic syndrome (aHUS) [1-3]. These associations have focused interest on the role of FH in pathogenesis and on the possibility of using pure FH for therapy [5,6]. A precedent for using FH in therapy is provided by aHUS, in which plasmatherapy has been used to replenish normal FH [7].

 The Y402H polymorphism in SCR7 of FH has attracted particular interest, because it is strongly associated with AMD. About 13 % of Caucasians are homozygous for the allele resulting in synthesis of the H402 protein and these individuals have a 6-fold increase in risk for AMD, compared to those homozygous for Y402, while the risk is increased 2.5-fold in heterozygotes [8]. AMD is the leading cause of blindness among the elderly in western societies with over 8 million affected in the U.S. alone [9]. Interestingly, intravitreal injection of pure human FH was shown to decrease laser-induced choroidal neovascularization (CNV) in rats, a model for CNV associated with AMD [10]. These findings suggest that many AMD patients might be treated locally or systemically with pure FH, in particular with preparations containing the Y402 variant [11].

 Much of the current interest in FH derives from reports that FH binds to surface proteins of pathogens, as first described for *Streptococcus pyogenes* M protein [12]. This coiled-coil protein has a hypervariable region (HVR) that exhibits extensive sequence variation among M proteins expressed by different strains, allowing the identification of ~200 M types [13]. We recently showed that some but not all M proteins bind FH and that FH binds solely to the HVR of an M protein [14]. In FH, the binding site for M protein is located in SCR7 and may overlap with the polymorphic site implicated in AMD [15-17].

 Here, we describe an affinity chromatography system, based on the HVR of the M5 protein, allowing efficient, single-step purification of FH from human serum. We also report that M proteins show differential binding to the Y402H polymorphic variants of FH. The M6 protein, in particular, shows preference for binding the Y402 variant, and a construct derived from this M protein enabled considerable enrichment of the Y402 protein, the FH variant that protects against AMD, from serum containing both variants. Thus, we have developed a simple method for single-step purification of FH. This method might enable the development of FH based therapies for AMD and other conditions with defective FH function.

## Results and Discussion

### Binding of FH to a construct derived from the HVR of the M5 protein

**Figure 1:**
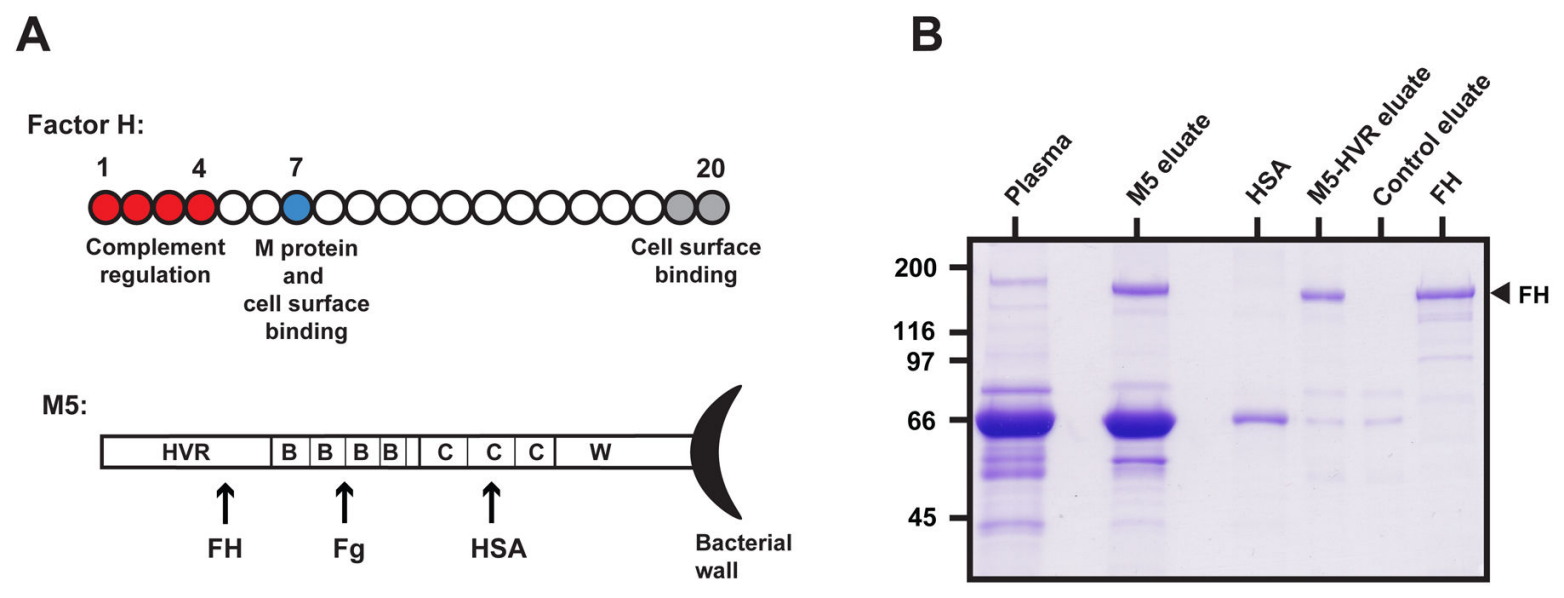
Affinity chromatography of plasma on M5 and the M5-HVR. (**A**) Proteins studied. Schematic representations of FH and *S. pyogenes* M5 protein. See text. (**B**) Affinity chromatography of human plasma (containing Y402 FH) on columns containing immobilized M5 or M5-HVR. Bound protein was eluted and analyzed by SDS-PAGE. A column without immobilized protein was used as control. Human plasma, HSA and pure FH were included for comparison. The location of FH is indicated. In the M5 eluate the major band in the ~50-60 kDa region was identified as HSA by mass spectrometry. Moreover, some weaker bands in this region were identified as Fg by western blot (not shown).

Among the 20 SCRs in FH, SCR1-4 are required for cofactor activity, SCR7 is implicated in binding to M proteins and to cellular surfaces [1,15,16] and SCR19-20 promote cell surface binding [1-3] (Figure 1A). Of note, SCR7 contains the Y402H polymorphism implicated in AMD.

 The M proteins that bind FH, such as M5, have an N-terminal HVR that mediates binding of FH, a B repeat region binding fibrinogen (Fg) and a C repeat region binding human serum albumin (HSA) (Figure 1A). These multiple binding properties indicated that an intact FH binding M protein would not be suitable for the purification of FH but focused interest on the use of isolated HVRs, which previously were found to bind FH [14]. To verify this prediction, we immobilized pure preparations of intact M5 and the M5-HVR in columns and applied whole human plasma (from an FH Y402 homozygote), followed by elution of bound proteins. As expected, both eluates contained FH, but the eluate from intact M5 also contained other proteins, including Fg and HSA. Of note, the FH eluted from the HVR column was virtually pure apart from small amounts of other proteins, also present in the eluate from a control column (Figure 1B).

**Figure 2:**
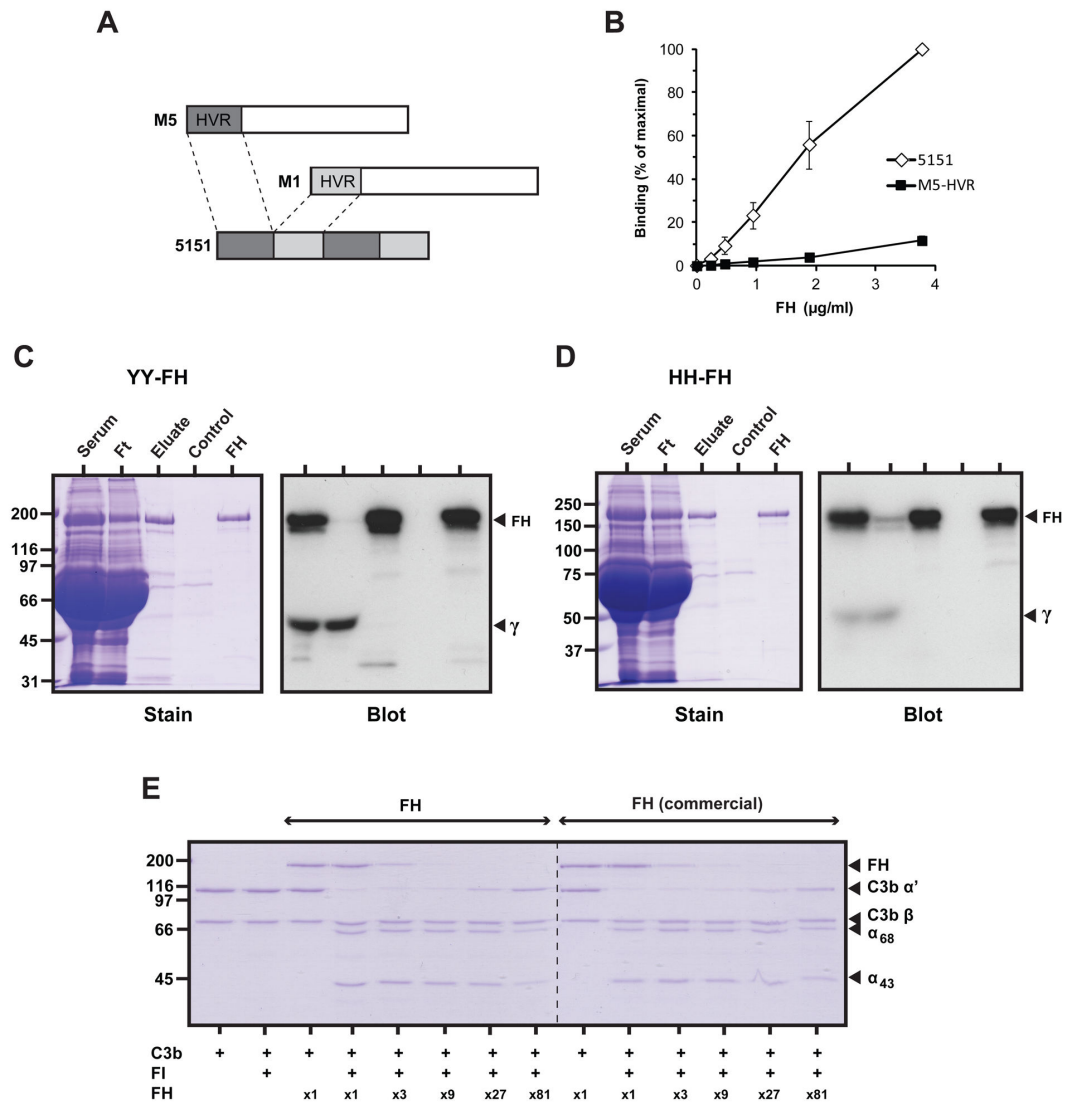
Affinity purification of FH on the 5151 construct. (**A**) Schematic representation of the 5151 construct. See text. (**B**) FH has higher affinity for 5151 than for the M5-HVR. The 5151 and M5-HVR proteins were immobilized and analyzed for ability to bind pure FH. The data represent mean values with SD from three experiments. (**C** and **D**) Affinity purification of FH on the 5151 construct and FH-depletion of human serum. Whole serum containing the Y402 protein (C) or the H402 protein (D) was applied to a column containing immobilized 5151. Bound protein was analyzed by SDS-PAGE (left panels) and western blot (right panels). The control represents the eluate from a column without immobilized 5151. Ft, flow-through. Pure FH was included as a reference. The blots were probed with sheep anti-FH. Bound antibodies were detected with radiolabeled protein G and autoradiography. Protein G also bound to the heavy chain of IgG (γ), as indicated. (**E**) Cofactor activity of purified Y402 FH compared to that of commercial FH. The analysis measured the ability of FH to act as a cofactor for FI in the degradation of the C3b α’ chain to 68 kDa and 43 kDa fragments of iC3b. The two FH proteins had similar activity when diluted, showing that they had similar cofactor activity.

 We hypothesized that a construct containing more than one copy of the M5-HVR might have superior FH-binding properties. A fusion protein containing two copies of the M5-HVR was therefore constructed. To separate the two binding sites we included two copies of the M1-HVR, which does not bind FH [14]. As a result, the final construct contained four HVRs in tandem and was designated 5151 (Figure 2A and S1A). The 5151 protein was dimerized via a C-terminal cysteine residue to promote coiled-coil formation and ligand binding [18] (Figure S1C, left). To compare the properties of the M5-HVR and 5151 (both dimerized), equimolar amounts were immobilized in microtiter wells and tested for FH binding. The 5151 construct showed considerably better FH binding than the M5-HVR (Figure 2B), focusing interest on 5151.

### Affinity purification of human FH on the 5151 construct

Single donor serum containing only the Y402 protein (“YY serum”) was used for affinity chromatography on a column containing 5151, and bound protein was eluted. FH dominated, as demonstrated by SDS-PAGE, western blot and mass spectrometry (Figure 2C and data not shown). Under the non-saturating conditions used, no FH was detected in the flow-through, implying that the serum was completely depleted of FH. Thus, the procedure allowed the purification of all FH from serum containing the Y402 protein. Similar results were obtained with another YY serum. Under saturating conditions, i.e. when an excess of serum was applied, ~0.9 mg of FH was recovered in a single run from a column prepared with 4 mg of 5151. 

 Similar analysis was performed with single donor serum containing only the H402 variant (“HH serum”). The flow-through from this column contained some FH, but also in this case most FH was retained on the column, implying that the 5151 construct can be used to purify the H402 variant of FH (Figure 2D).

 Theoretically, the 42 kDa FH splice variant FH-like protein 1 (FHL-1) consisting of SCRs 1-7 of FH [15] would be expected to bind the 5151 construct. We have, however, not been able to detect a 42 kDa band reacting with the anti FH antibody (Figure 2C and D), while a weak ~35 kDa band was observed in some gels, possibly representing a degradation product of FH.

 To investigate if the purified FH retained its ability to act as a cofactor for factor I (FI) in the degradation of C3b, we compared the pure Y402 protein with commercial FH. Various amounts of FH were mixed with FI and C3b and the FH-dependent cleavage of C3b was analyzed by SDS-PAGE. In this analysis, the purified Y402 FH was as active as commercial FH (Figure 2E). Similar results were obtained with H402 FH purified on a 5151 column (not shown).

 Together, our data indicate that the 5151 construct may be used for simple, single-step purification of FH retaining its biological activity. We estimate that this FH had a purity of >90%, which may be sufficient for many applications. For example, hereditary angioedema is treated with plasma-derived C1 inhibitor having an estimated purity of ≥90% [19,20]. Of note, a 5151 column was very stable, surviving regeneration 50 times over a period of 2 years without apparent diminution of binding activity. 

### M proteins differ in relative affinity for the Y402 and H402 variants

It has been reported that the Y402H polymorphism has a strong effect on binding of FH to the M6 protein [17]. To analyze whether this finding applies to other FH-binding M proteins, we compared the three M proteins for which the FH-binding HVR has been characterized, the M5, M6 and M18 proteins [14]. These M proteins were analyzed for ability to bind the Y402 and H402 proteins (purified by methods previously described [21]).

**Figure 3:**
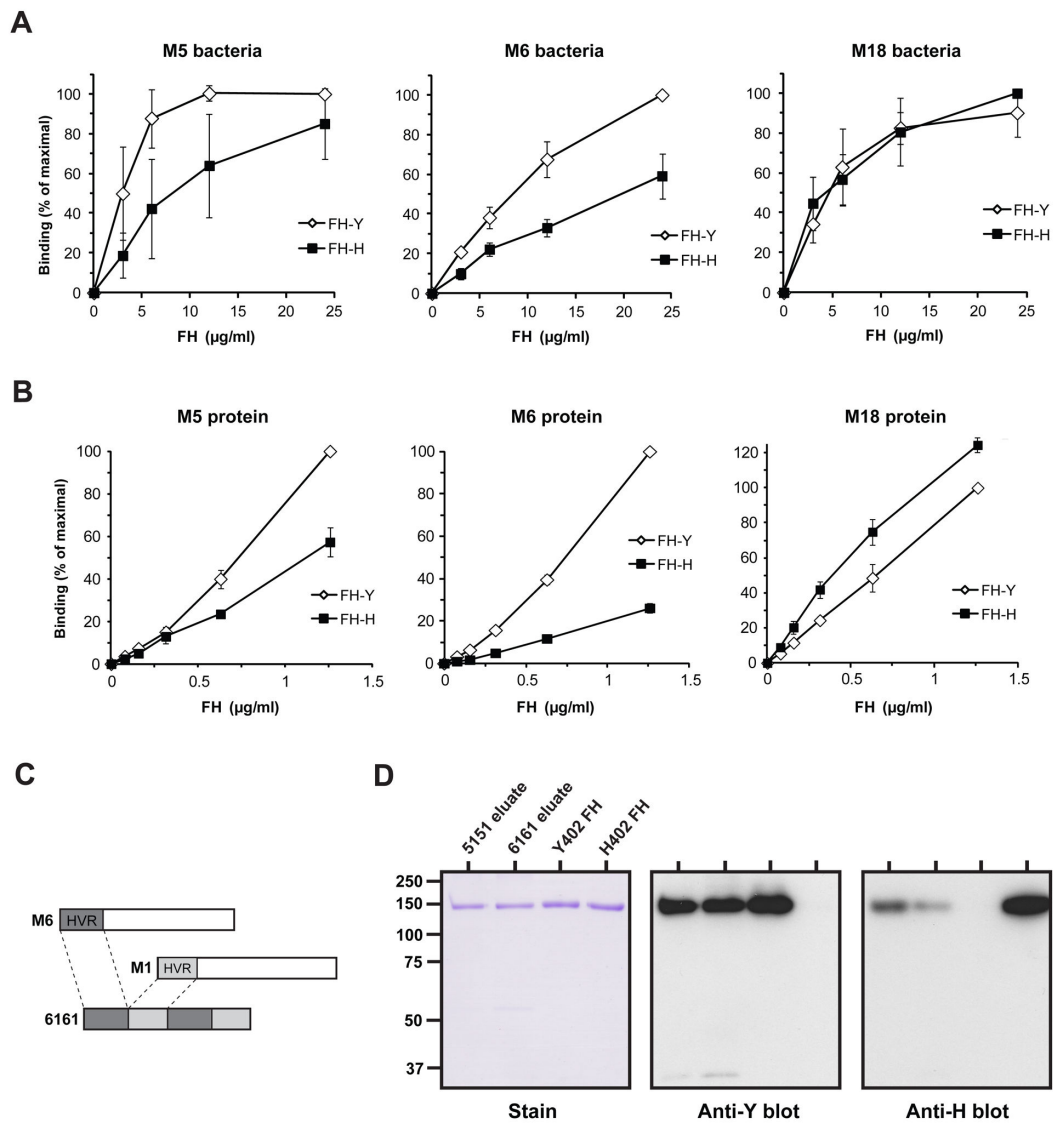
Differential binding of Y402 and H402 FH to different M proteins: enrichment of the Y402 protein on an M6 derived construct. (**A**) Binding of pure Y402 and H402 FH to *S. pyogenes* M5, M6 and M18 bacteria. The pure FH proteins were added at the concentrations indicated. Bound FH was detected with sheep anti-FH and radiolabeled protein G. The binding to M-negative control strains was low (not shown). (**B**) Differential binding of the Y402 and H402 proteins to pure M5, M6 and M18 immobilized in microtiter wells. The FH proteins were added at the concentrations indicated. Bound FH was detected with sheep anti-FH and radiolabeled protein G. The data in A and B represent mean values with SD from three experiments. Two-way ANOVA analysis was performed on the binding values corresponding to the highest concentrations of FH corrected for the difference between individual experiments (not set to 100 % as in the graph) (n=3). The differences in binding to Y402 and H402 FH were found to be significant with P<0.001 for M5, P<0.001 for M6 and P=0.004 for M18. (**C**) Schematic representation of the 6161 construct. See text. (**D**) Affinity purification of FH on the 6161 and 5151 constructs. Serum containing Y402 and H402 FH (from a heterozygote) was applied to columns with immobilized 6161 or 5151. Bound protein was eluted and analyzed by SDS-PAGE (left panel) and western blot (middle and right panels). The blots were probed with mAbs specific for Y402 or H402 FH, as indicated. Pure Y402 and H402 FH were included as controls.

 Binding was first analyzed with whole bacteria expressing the M proteins (Figure 3A). The strains expressing M5 or M6 showed better binding of the Y402 protein, while no difference was observed for the M18 strain. Similar results were observed in studies of pure M proteins, immobilized in microtiter wells (Figure 3B). The greatest difference in binding for the FH variants was observed for the M6 protein which bound Y402 much more strongly than H402, with a similar but smaller difference in binding to M5. Moreover, a small difference in binding was observed also for M18, with a preference for the other FH variant, H402. Thus, the studies of whole bacteria and pure M proteins gave similar results and demonstrate that the effect of the Y402H polymorphism on binding differs among M proteins.

### Use of the HVR in M6 for enrichment of the Y402 variant

The variant-selective properties of M6 suggested that the HVR of this protein might be used for selective purification of the Y402 protein from serum containing both FH variants, e.g. from serum or plasma pools. Following the procedure employed in the M5 system, we therefore prepared a fusion construct with two copies of the M6-HVR and two copies of the non-binding M1-HVR, resulting in a dimerized polypeptide designated 6161 (Figure 3C, S1B and S1C). Serum from a heterozygote (“YH serum”) was applied to a 6161 column and bound material was eluted. For comparison, similar analysis was performed with immobilized 5151.

 The eluates from the 5151 and 6161 columns were analyzed by SDS-PAGE and western blot, using pure Y402 and H402 proteins as controls (Figure 3D). The blots were analyzed with mAbs specific for the Y402 or the H402 protein [21,22]. Pure FH was eluted from both columns and this material reacted with the two mAbs. However, the strength of the blotting signals indicated that the material eluted from the 6161 column was significantly enriched for the Y402 variant. Quantitative ELISA confirmed this conclusion. The starting material contained 51% Y402 protein, but in eluates from the 6161 column the proportion of Y402 protein had increased to 71%. Identical results were obtained twice. For the FH eluted from the 5151 column, the Y402 protein represented 63%, i.e. a modest enrichment of Y402 FH was obtained also on this column, probably reflecting incomplete binding of the H402 variant to 5151 (Figure 2D).

 We hypothesized that application of saturating amounts of serum to a 6161 column would cause further enrichment of the Y402 protein, because the column would preferentially bind the Y402 protein. To test this, the amount of serum applied was increased 30-fold; the proportion of Y402 FH in the eluate indeed increased to 79%. Identical results were obtained with two sera. Thus, considerable enrichment of the Y402 protein could be achieved in this single step.

## Concluding Remarks

The current interest in FH as a therapeutic agent emphasizes that a simple purification method is needed. The method described here allows the efficient recovery of virtually pure FH in a single step, while traditional purification methods involve several steps with concurrent loss of material [23]. Our method is based on the recent demonstration that the HVR of some streptococcal M proteins specifically binds human FH [14]. Thus, the method exploits the exquisite binding specificity of a bacterial virulence factor. The value of such methods is demonstrated by the classical example of staphylococcal protein A, which is used for the purification of IgG [24]. Moreover, certain regions in streptococcal M proteins can be employed for the purification of human IgA and human C4b-binding protein [25,26].

 Of note, the M protein-derived constructs can be produced economically and in large amounts, potentially allowing large-scale purification of FH, which would be needed for therapy. Moreover, all available data indicate that the constructs are stable and survive extensive use. This is in contrast to the polyclonal and monoclonal antibodies that have been successfully employed for small-scale FH purification [21,27,28], for which the production costs probably would constrain large-scale FH preparation.

 An additional advantage of the M protein derived system described here is that it may allow enrichment of the Y402 variant of FH from pooled plasma, potentially facilitating the development of an FH-based therapy for AMD. Furthermore, the method may be adapted for the purification of FH from other sources, e.g. recombinantly produced FH [5]. In summary, our studies show that the highly specific binding between FH and certain M proteins can be used to develop a simple and robust FH purification method.

## Materials and Methods

### Bacterial strains and media

The *S. pyogenes* M5, M6 and M18 strains and their M-negative mutants have been described ([14] and references therein). These strains were grown as described [14] in Todd-Hewitt broth supplemented with 0.2 % yeast extract and the protease inhibitor E64. *Escherichia coli* XL1 Blue was used for cloning and strain BL21 for protein production. *E. coli* was grown with shaking at 37°C in LB, supplemented when needed with 100 μg/ml ampicillin. 

### Human proteins, serum and plasma

FI, C3b, and control FH were from Complement Technology. The control FH contains both the Y402 and H402 variants. The preparations of pure Y402 and H402 FH used in Figure 3A, 3B, and 3D were isolated as described [21]. Outdated, anonymized, human citrated plasma was purchased from Lund University Hospital Blood Centre, with permission (2012:04). Serum was derived from the citrated plasma as described [14]. Before use, the analyzed plasma and serum samples were heat-inactivated for 30 min at 56°C, to block complement activation. Specific mAbs were used to type the samples with regard to the Y402H polymorphism, as described [21,22].

### Pure recombinant M proteins, HVRs and fusion proteins

All recombinant proteins were derived from GST-fusions (GE Healthcare). The intact M1, M5, M6, and M18 proteins, and the HVRs of M1 and M5 have been described [14,29]. The recombinant fusion protein 5151 (Figure 2A) consists of amino acid residues 1-117 of M5, 4-89 of M1, 1-117 of M5 and 4-91 of M1 (numbering in processed form of the proteins) (Figure S1A). The 5151 DNA coding sequence was constructed by overlap PCR in several steps using standard methodology. The final PCR product was cloned into plasmid pGEX-6P-2 (GE Healthcare) cleaved with BamHI and EcoRI. The sequence of the final construct was confirmed. The recombinant protein 6161 consists of amino acid residues 1-129 of M6, 8-88 of M1, 1-129 of M6 and 8-91 of M1 (Figure S1B). The construct encoding 6161 was created by gene synthesis and cloned into pGEX-6P-2 as described for 5151 (GenScript). To allow dimerization, a C-terminal cysteine not present in the intact M protein was added to 5151, 6161, M5-HVR and M1-HVR. For this purpose, a cysteine codon was added in the corresponding DNA constructs. After removal of the GST tag, the protein constructs were dimerized as described [18]. Analysis of the 5151 and 6161 proteins by SDS-PAGE under reducing and non-reducing conditions showed good but not complete dimerization (Figure S1C). Of note, the M protein HVRs were dimerized as described for 5151 and 6161, while intact M proteins dimerize spontaneously.

### Antisera

Sheep anti-human FH IgG (The Binding Site) was used to detect total human FH. The monoclonal mouse IgG_1_ antibodies MBI-6 and MBI-7 were used to detect Y402 and H402 FH, respectively [21,22]. Goat anti-mouse IgG horseradish peroxidase conjugated antibody (Thermo Scientific) was used for detecting mouse IgG in western blots.

### FH binding assays with immobilized pure ligands and whole bacteria

The tests with pure ligands reported in Figure 2B and 3B were performed essentially as described [14]. For Figure 2B, 0.1 μg 5151 or an equimolar amount of the M5-HVR were immobilized in microtiter wells. The M1-HVR was used as negative control. For Figure 3B, 0.1 μg of pure M5, M6 or M18 was immobilized, with M1 as negative control. In Figure 3B, the buffers (except those used for coating and blocking) were diluted × 3 in distilled water, a procedure that reduced background binding. Pure FH was added, as indicated, and bound FH was detected with sheep anti-FH (diluted × 500) and radiolabeled protein G. Binding was calculated in percent of maximal protein G binding (Figure 2B) or in percent of maximal protein G binding in the test with Y402 FH (Figure 3B). Binding to control proteins was very low and has been subtracted. The binding tests with whole bacteria (Figure 3A) were performed essentially as described [14]. Briefly, samples containing 10^8^ cfu of washed bacteria were incubated with the indicated amount of Y402 or H402 FH for 1 h in a volume of 100 μl. Bound FH was quantified by the addition of sheep anti-human FH and bound anti-FH was detected with radiolabeled protein G.

### Affinity chromatography

For Figure 1B, pure recombinant M5 or M5-HVR (600 μg) was used for coupling to 1 ml HiTrap NHS-activated HP columns (GE Healthcare). For the generation of a control column, reactive groups were inactivated with ethanolamine. Before use, columns were equilibrated with 10 volumes of PBS. Human plasma (1.5 ml, diluted × 3 in PBS), containing Y402 FH, was applied slowly at 4°C and the columns were washed with 10 volumes of PBS. Bound protein was eluted in 5 ml 6 M guanidine-HCl, dialysed against PBS and concentrated 10-fold before analysis.

 For the tests reported in Figure 2C, 2D, and 3D, the 5151 protein (4 mg) or the 6161 protein (2 mg) was coupled to columns, as indicated. The serum applied (1.5 ml) had been typed with regard to the Y402H polymorphism, as indicated. Subsequent steps were performed as described for Figure 1B.

### Other methods

Western blot and detection of bound IgG with radiolabeled protein G was performed as described [30]. Western blots with anti-FH mAbs (Figure 3D) were performed essentially as described [21,22]. Quantitative determination of Y402 and H402 FH was performed according to [22]. FH concentration was determined spectrophotometrically, using an extinction coefficient at 280 nm of 1.97 [4].

 Assays for FH cofactor activity were performed essentially as described [31], with the exception that C3b was not radiolabeled. Briefly, 20 µl reactions (final volume) with final concentrations of 40 µg/ml C3b and 4 µg/ml FI were prepared in PBS (diluted × 3). Pure FH was added to a final concentration of 12.6 µg/ml, or using different 3-fold dilutions of this concentration, as indicated. After 2 h at 37°C the reactions were terminated by the addition of an equal volume of SDS-PAGE sample buffer and the samples were analyzed by SDS-PAGE.

### Statistical analysis

Results from FH binding assays (Figure 2B, 3A and 3B) are presented as mean values with SD from three independent determinations. For Figure 3B, two-way ANOVA analysis was performed on the binding values (normalized to the added radioactivity) corresponding to the highest FH concentrations, corrected for the difference between the individual experiments.

## Supporting Information

Figure S1 **The 5151 and 6161 fusion proteins.** (**A**) Sequence of the 5151 protein. The underlined sequences were derived from M5, while the other parts were derived from M1. (**B**) Sequence of the 6161 protein. The underlined sequences were derived from M6, while the other parts were derived from M1. For both 5151 and 6161, a C-terminal cysteine residue, not present in the intact M proteins, was added to allow dimerization. (**C**) Pure dimerized recombinant 5151 (left panel) and 6161 (right panel) was analyzed by SDS-PAGE. In each panel, the sample to the left was run under reducing conditions and the sample to the right was run under non-reducing conditions, allowing the detection of dimerized 5151 and 6161.
(TIF)

## References

[1] Józsi M, Zipfel PF (2008) Factor H family proteins and human diseases. Trends Immunol 29: 380-387. doi:10.1016/j.it.2008.04.008. PubMed: 18602340. 18602340

[2] Rodríguez de Córdoba S, Goicoechea de Jorge E (2008) Translational mini-review series on complement factor H: genetics and disease associations of human complement factor H. Clin Exp Immunol 151: 1-13. PubMed: 18081690. 18081690 10.1111/j.1365-2249.2007.03552.x PMC2276932

[3] Makou E, Herbert AP, Barlow PN (2013) Functional anatomy of complement factor H. Biochemistry 52: 3949-3962. doi:10.1021/bi4003452. PubMed: 23701234. 23701234

[4] Sofat R, Mangione PP, Gallimore JR, Hakobyan S, Hughes TR et al. (2013) Distribution and determinants of circulating complement factor H concentration determined by a high-throughput immunonephelometric assay. J Immunol Methods 390: 63-73. doi:10.1016/j.jim.2013.01.009. PubMed: 23376722. 23376722

[5] Schmidt CQ, Slingsby FC, Richards A, Barlow PN (2011) Production of biologically active complement factor H in therapeutically useful quantities. Protein Expr Purif 76: 254-263. doi:10.1016/j.pep.2010.12.002. PubMed: 21146613. 21146613 PMC4067574

[6] Ricklin D, Lambris JD (2013) Complement in immune and inflammatory disorders: therapeutic interventions. J Immunol 190: 3839-3847. doi:10.4049/jimmunol.1203200. PubMed: 23564578. 23564578 PMC3623010

[7] Loirat C, Frémeaux-Bacchi V (2011) Atypical hemolytic uremic syndrome. Orphanet J Rare Dis 6: 60. doi:10.1186/1750-1172-6-60. PubMed: 21902819. 21902819 PMC3198674

[8] Thakkinstian A, Han P, McEvoy M, Smith W, Hoh J et al. (2006) Systematic review and meta-analysis of the association between complement factor H Y402H polymorphisms and age-related macular degeneration. Hum Mol Genet 15: 2784-2790. doi:10.1093/hmg/ddl220. PubMed: 16905558. 16905558

[9] Jager RD, Mieler WF, Miller JW (2008) Age-related macular degeneration. N Engl J Med 358: 2606-2617. doi:10.1056/NEJMra0801537. PubMed: 18550876. 18550876

[10] Kim SJ, Kim J, Lee J, Cho SY, Kang HJ et al. (2013) Intravitreal human complement factor H in a rat model of laser-induced choroidal neovascularisation. Br J Ophthalmol 97: 367-370. doi:10.1136/bjophthalmol-2012-302307. PubMed: 23258212. 23258212

[11] Troutbeck R, Al-Qureshi S, Guymer RH (2012) Therapeutic targeting of the complement system in age-related macular degeneration: a review. Clin Experiment Ophthalmol 40: 18-26. doi:10.1111/j.1442-9071.2011.02581.x. PubMed: 22304025. 22304025

[12] Blom AM, Hallström T, Riesbeck K (2009) Complement evasion strategies of pathogens - acquisition of inhibitors and beyond. Mol Immunol 46: 2808-2817. doi:10.1016/j.molimm.2009.04.025. PubMed: 19477524. 19477524

[13] Steer AC, Law I, Matatolu L, Beall BW, Carapetis JR (2009) Global *emm* type distribution of group A streptococci: systematic review and implications for vaccine development. Lancet Infect Dis 9: 611-616. doi:10.1016/S1473-3099(09)70178-1. PubMed: 19778763. 19778763

[14] Gustafsson MC, Lannergård J, Nilsson OR, Kristensen BM, Olsen JE et al. (2013) Factor H binds to the hypervariable region of many *Streptococcus* *pyogenes* M proteins but does not promote phagocytosis resistance or acute virulence. PLOS Pathog 9: e1003323. 23637608 10.1371/journal.ppat.1003323 PMC3630203

[15] Kotarsky H, Hellwage J, Johnsson E, Skerka C, Svensson HG et al. (1998) Identification of a domain in human factor H and factor H-like protein-1 required for the interaction with streptococcal M proteins. J Immunol 160: 3349-3354. PubMed: 9531294. 9531294

[16] Blackmore TK, Fischetti VA, Sadlon TA, Ward HM, Gordon DL (1998) M protein of the group A *Streptococcus* binds to the seventh short consensus repeat of human complement factor H. Infect Immun 66: 1427-1431. PubMed: 9529063. 9529063 10.1128/iai.66.4.1427-1431.1998 PMC108070

[17] Yu J, Wiita P, Kawaguchi R, Honda J, Jorgensen A et al. (2007) Biochemical analysis of a common human polymorphism associated with age-related macular degeneration. Biochemistry 46: 8451-8461. doi:10.1021/bi700459a. PubMed: 17580967. 17580967

[18] Morfeldt E, Berggård K, Persson J, Drakenberg T, Johnsson E et al. (2001) Isolated hypervariable regions derived from streptococcal M proteins specifically bind human C4b-binding protein: implications for antigenic variation. J Immunol 167: 3870-3877. PubMed: 11564804. 11564804 10.4049/jimmunol.167.7.3870

[19] Zuraw BL, Busse PJ, White M, Jacobs J, Lumry W et al. (2010) Nanofiltered C1 inhibitor concentrate for treatment of hereditary angioedema. N Engl J Med 363: 513-522. doi:10.1056/NEJMoa0805538. PubMed: 20818886. 20818886

[20] Farkas H, Varga L (2012) Human plasma-derived, nanofiltered, C1-inhibitor concentrate (Cinryze), a novel therapeutic alternative for the management of heriditary angioedema resulting from C1-inhibitor deficiency. Biol Ther 2: 1-17. doi:10.1007/s13554-012-0001-6. 24490128 PMC3906706

[21] Hakobyan S, Harris CL, Tortajada A, Goicochea de Jorge E, García-Layana A et al. (2008) Measurement of factor H variants in plasma using variant-specific monoclonal antibodies: application to assessing risk of age-related macular degeneration. Invest Ophthalmol Vis Sci 49: 1983-1890. doi:10.1167/iovs.07-1523. PubMed: 18436830. 18436830

[22] Hakobyan S, Tortajada A, Harris CL, Rodríguez de Córdoba S, Morgan BP (2010) Variant-specific quantification of factor H in plasma identifies null alleles associated with atypical hemolytic uremic syndrome. Kidney Int 78: 782-788. doi:10.1038/ki.2010.275. PubMed: 20703214. 20703214 PMC3252682

[23] Brandstätter H, Schulz P, Polunic I, Kannicht C, Kohla G et al. (2012) Purification and biochemical characterization of functional complement factor H from human plasma fractions. Vox Sang 103: 201-212. doi:10.1111/j.1423-0410.2012.01610.x. PubMed: 22497541. 22497541

[24] Forsgren A, Sjöquist J (1966) "Protein A" from S. aureus. I. Pseudo-immune reaction with human gamma-globulin. J Immunol 97: 822-827. 4163007

[25] Sandin C, Linse S, Areschoug T, Woof JM, Reinholdt J et al. (2002) Isolation and detection of human IgA using a streptococcal IgA-binding peptide. J Immunol 169: 1357-1364. PubMed: 12133959. 12133959 10.4049/jimmunol.169.3.1357

[26] Persson J, Lindahl G (2005) Single-step purification of human C4b-binding protein (C4BP) by affinity chromatography on a peptide derived from a streptococcal surface protein. J Immunol Methods 297: 83-95. doi:10.1016/j.jim.2004.11.024. PubMed: 15777933. 15777933

[27] Pangburn MK, Schreiber RD, Müller-Eberhard HJ (1977) Human complement C3b inactivator: isolation, characterization, and demonstration of an absolute requirement for the serum protein β1H for cleavage of C3b and C4b in solution. J Exp Med 146: 257-270. doi:10.1084/jem.146.1.257. PubMed: 301546. 301546 PMC2180748

[28] Sim RB, Day AJ, Moffatt BE, Fontaine M (1993) Complement factor I and cofactors in control of complement system convertase enzymes. Methods Enzymol 223: 13-35. doi:10.1016/0076-6879(93)23035-L. PubMed: 8271948. 8271948

[29] Lannergård J, Gustafsson MC, Waldemarsson J, Norrby-Teglund A, Stålhammar-Carlemalm M et al. (2011) The hypervariable region of *Streptococcus* *pyogenes* M protein escapes antibody attack by antigenic variation and weak immunogenicity. Cell Host Microbe 10: 147-157. doi:10.1016/j.chom.2011.06.011. PubMed: 21843871. 21843871

[30] Stålhammar-Carlemalm M, Stenberg L, Lindahl G (1993) Protein Rib: a novel group B streptococcal cell surface protein that confers protective immunity and is expressed by most strains causing invasive infections. J Exp Med 177: 1593-1603. doi:10.1084/jem.177.6.1593. PubMed: 8496678. 8496678 PMC2191029

[31] Harris CL (2000) Functional assays for complement regulators. In: Morgan BP Complement methods and protocols. Totowa, NJ: Humana Press, Inc. pp. 83-101. 10.1385/1-59259-056-X:83 10857104

