# Supplementary figures and images for: Affinity Purification of Human Factor H on Polypeptides Derived from Streptococcal M Protein: Enrichment of the Y402 Variant
Source: PLoS One. 2013 Nov 21;8(11):e81303. doi: 10.1371/journal.pone.0081303 (PMC3836803; doi:10.1371/journal.pone.0081303)

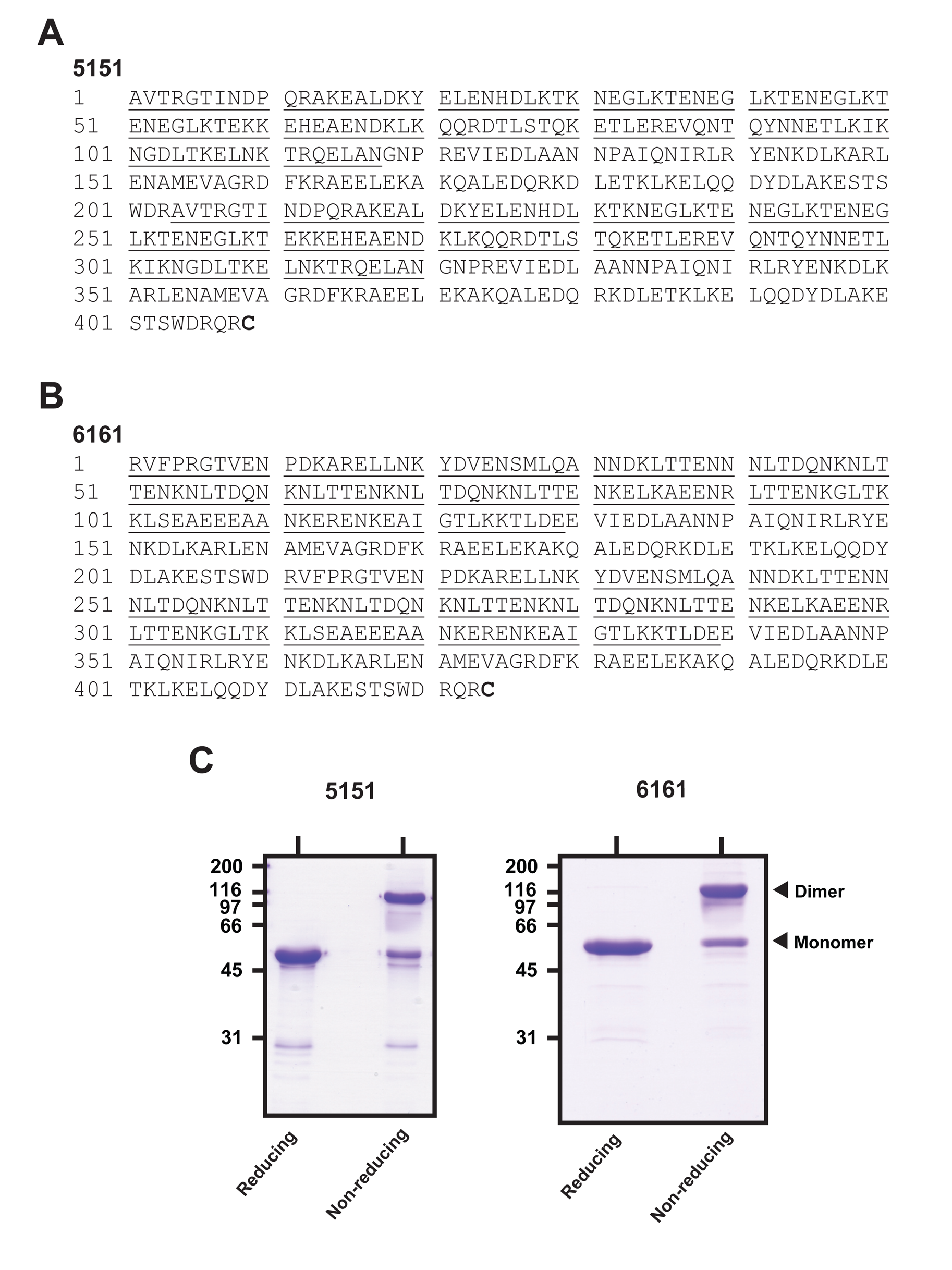

Supplement: Figure S1 — The 5151 and 6161 fusion proteins. (A) Sequence of the 5151 protein. The underlined sequences were derived from M5, while the other parts were derived from M1. (B) Sequence of the 6161 protein. The underlined sequences were derived from M6, while the other parts were derived from M1. For both 5151 and 6161, a C-terminal cysteine residue, not present in the intact M proteins, was added to allow dimerization. (C) Pure dimerized recombinant 5151 (left panel) and 6161 (right panel) was analyzed by SDS-PAGE. In each panel, the sample to the left was run under reducing conditions and the sample to the right was run under non-reducing conditions, allowing the detection of dimerized 5151 and 6161. (TIF) [file pone.0081303.s001.tif]
